# Structure and functional divergence of PIP peptide family revealed by functional studies on PIP1 and PIP2 in *Arabidopsis thaliana*

**DOI:** 10.3389/fpls.2023.1208549

**Published:** 2023-11-24

**Authors:** Xiao-song Yu, Hong-run Wang, Fei-fan Lei, Rui-qi Li, Hui-peng Yao, Jin-bo Shen, Noor-ul Ain, Yi Cai

**Affiliations:** ^1^ College of Life Sciences, Sichuan Agricultural University, Yaan, Sichuan, China; ^2^ State Key Laboratory of Subtropical Silviculture, Zhejiang A&F University, Hangzhou, Zhejiang, China; ^3^ Guangdong Laboratory for Lingnan Modern Agriculture, Genome Analysis Laboratory of the Ministry of Agriculture, Agricultural Genomics Institute at Shenzhen, Chinese Academy of Agricultural Sciences, Shenzhen, Guangdong, China

**Keywords:** Arabidopsis, PIP, functional divergence, growth, pathogens resistance

## Abstract

PAMP-induced secreted peptide (PIP), one of the small post-translationally modified peptides (PTMPs), plays a crucial role in plant development and stress tolerance. However, little is known about functional divergence among this peptide family. Here, we studied the evolution of the PIP family in 23 plant species (10 monocotyledons and 13 dicotyledons from 7 families) and their functional divergence in Arabidopsis. A total of 128 putative PIP precursors were identified and classified into two subfamilies through phylogenetic analysis. Functional studies on AtPIP1 which represents Clade I family and AtPIP2 which represents Clade II family have shown that AtPIP2 displayed stronger immunity induction activity but weaker root growth inhibition than AtPIP1 in Arabidopsis. Transcriptome analysis of Arabidopsis seedlings treated with AtPIP1 and AtPIP2 showed that differential genes for both polypeptides were significantly enriched in similar plant defense pathways. However, Co-expression and Protein-protein interaction (PPI) analysis showed that the functions of AtprePIP2 co-expressed genes were more enriched in plant defense pathways than AtprePIP1. Molecular docking results show that AtPIP1 binds to RLK7 receptor with a more stable free energy and less binding area than AtPIP2, while hydrogen bond transfer occurs at the SGP motif position. The above results suggest that the PIP family have undergone functional divergence during evolution. Collectively, this work illustrates the relationship between PIP structure and function using Arabidopsis PIP as an example, and provides new insights into the current understanding between growth inhibition and immune responses which may be correlated but not fully coupled.

## Introduction

Plants have evolved sophisticated sensory and defense systems to defend themselves against pathogens. These systems are regulated by complex networks formed by proteins and hormones and are associated with changes in gene expression (Buscaill and Rivas, 2014). Among them, small secretory peptide (SSPs) plays an important role in regulating development and defending against pathogens through ligand-receptor interactions (Murphy et al., 2012; Marmiroli and Maestri, 2014; Gust et al., 2017). As crucial components of intercellular communication, they interfere with signaling and response pathways or display direct antimicrobial activity. Based on their general characteristics, more than 1000 putative peptides have been identified in Arabidopsis using bioinformatics tools (Lease and Walker, 2010). In general, peptides are characterized by having an N-terminal signal peptide (SP) with a variable region in the middle and a conserved C-terminus containing the active peptide (Matsubayashi, 2014). They are initially translated as precursor peptides and directed to the secretory pathway by SP, during which they undergo additional specific modifications required for peptide-receptor binding (Matsubayashi, 2014; Chen et al., 2020a), namely small post-translationally modified peptides (PTMPs), such as RGF (Matsuzaki et al., 2010), CLE (Fletcher, 2020), IDA (Butenko et al., 2003), etc. These peptides coordinate the initial environmental stress response followed by cell to cell signaling through ligand-receptor interactions (Chen et al., 2020a). Thus, peptide ligands act as important mediators of intercellular communication during plant growth and stress responses (Matsubayashi and Sakagami, 2006; Matsubayashi, 2014).

In Arabidopsis, pathogen-associated molecular pattern (PAMP)-induced AtPIP1 and AtPIP2 were identified by activating receptor-like kinase 7 (RLK7) (Hou et al., 2014). PIPs, like other SSPs, are produced as precursor peptides with an N-terminal SP. After entering the secretory pathway, the SP is removed and the resulting precursor peptide is further processed into a 15-25 amino acid mature peptide. The expression of precursor gene of AtPIP1 and AtPIP2 was significantly upregulated under the treatment of exogenous PAMPs such as flagellin and chitin and trigger PAMP-triggered immunity (PTI) responses, including ROS bursts and regulation of defense genes expression (Hou et al., 2014). It has also been shown that AtPIP3 regulates plant immunity by modulating crosstalk between salicylate and jasmonate signaling pathways (Najafi et al., 2020). In addition, TOLS2/PIP-Like3 peptide, which inhibits lateral root initiation through interaction with RLK7 (Toyokura et al., 2019). Subsequently, PIP1 was identified in potatoes (*Solanum tuberosum*) and is involved in enhancing plant resistance against *potato virus Y* (PVY) infection (Combest et al., 2021). Indeed, homologous prePIP proteins are present in many species of dicotyledonous and monocotyledonous plants, such as soybean, grape, maize and rice, suggesting that PIP is widespread and functional in many species (Hou et al., 2014). Compared to phylogenetic studies on the more versatile small secreted peptides RGF and CLE (Strabala et al., 2014; Furumizu and Sawa, 2021), less is known about PIP family.

The Arabidopsis PIP family consists of 11 members. Some of these are transcriptionally induced by biotic and/or abiotic stresses (Hou et al., 2014; Vie et al., 2015). All members of the prePIP family have characteristics of post-translational modifications of secretory peptide precursors: the signal peptide, a highly conserved SGP-rich C-terminus motif, and a variable region in between (Matsubayashi, 2011). Therefore, they are called the SGP-rich peptide superfamily together with CLV3/CLE (Ito et al., 2006; Kondo et al., 2006), IDA (Butenko et al., 2003), CEP1 (Ohyama et al., 2008) and PEP1 peptides (Huffaker et al., 2006). A major feature of this family is that proline (P) in the SGP motif is a potential target for hydroxylation. AtPIP1 contains one SGP, while AtPIP2 contains two SGPs, but differences in activity and function between them are obscure.

Therefore, in this study, we investigated the precursor structures and evolutionary patterns of 23 important and representative plant species selected from the Ensemble plant database, and finally curated the representative sequences of the two major PIP families. Two representative sequences were experimentally found to produce functional divergence in Arabidopsis, and the reasons for this were further elaborated by molecular docking, gene co-expression, and RNA-seq. This study will provide a basis for further study of PIP and a valuable reference for further functional analysis of PIP in plant development and immunity.

## Materials and methods

### Plant materials

All the plant materials i.e., wild-type Arabidopsis, transcriptional reporter lines (DR5::GFP and WOX5::GFP) and mutants *rlk7* (SALK_083114) used in this study were derived from *Arabidopsis thaliana* Columbia ecotype (*Arabidopsis thaliana*, Col-0). Four-week-old Arabidopsis plants were grown in nutrient soils (soil: vermiculite: perlite = 1:1:1) in a growth room at 20-23 °C, 60% humidity and 12 h light. All plants were grown in a short-day artificial climate chamber (12 h of light (140 μmol·m^-2^·s^-1^), 22°C and 60% humidity).

### Peptide synthesis

All peptides of 95% purity were synthesized by GenScript Company (Nanjing, China), including AtPIP1-n (RLASGPSPRGRGH), AtPIP2-n (VKHSGPSPSGPGH), AtPIP1 (RLASGP^Hpy^SPRGRGH) and AtPIP2 (VKHSGP^Hpy^SPSGP^Hpy^GH).

### Root growth inhibition assay

Arabidopsis seeds were surface sterilized for 5min in 75% alcohol and washed three times with sterile water. The seeds were planted in 1/2 MS medium (0.8% agar, 1% sucrose, pH 5.7) with or without 1 μM peptides and vernalized at 4°C for 2 days, and then moved to an artificial climate chamber (12 h of light (140 μmol·m^-2^·s^-1^), 22°C, 60% humidity) for vertical incubation. Seedling root length was measured at 7th day. Three biological replicates, each with three technical repeats, were conducted for each experiment.

### Observation of transgenic plants with GFP signal

Transgenic plant seeds expressing DR5::GFP and WOX5::GFP were sterilized, vernalized and grown vertically for 11 days in 1/2 MS plates with or without 1 μM peptides (AtPIP1-n, AtPIP2-n, AtPIP1 and AtPIP2). GFP-labeled seedling root tip cells were excited at 488 nm and the emitted light fluorescence signal was detected at 495 nm to 550 nm. Images were obtained using an OLYMPUS-FV10-MCPUS (OLYMPUS CORPORATION, Tokyo, Japan) confocal laser scanning microscope. Quantification of GFP fluorescent signal was performed using ImageJ software. Three biological replicates, each with three technical repeats, were conducted for each experiment.

### ROS measurement

A luminol-based assay was used to quantify ROS in processed leaves (Wang et al., 2010). The third or fourth pair of true leaves from four-week-old soil-grown Arabidopsis plants were excised into leaf discs (3 mm diameter). Leaf discs were incubated in 30 mL water for 12 h in a 96-well plate followed by sequential addition of 99 µL of the reaction solution (200 µM luminol, 2 µg/mL peroxidase) and 100 nM peptide. Each well was measured once per minute for a total of 60 min. ROS production was indicated as means of relative light units (RLU) and plotted using Excel 2019. Six biological replicates, each with four technical repeats, were conducted for each experiment.

### Callose deposition

The method for detection of callose deposition was slightly modified from the protocol of Clay et al. (2009). Four-week-old Arabidopsis leaves were injected with peptides (H_2_O as control) and treated overnight (approximately 12 h). Leaves were fixed in FAA solution (10% formaldehyde, 5% acetic acid, 50% ethanol) for 24 h and transferred to anhydrous ethanol for 6h decolorization, followed by incubation in 50% ethanol for 30 min. Ethanol was removed and 67 mM K_2_HPO_4_ (pH 12) was added and incubated for 30 min. The K_2_HPO_4_ solution was removed and the staining solution (0.01% aniline blue in 67 mM K_2_HPO_4_, pH 12) was added and incubated for 1 h. Callose deposition was observed by microscopic UV light and photographed by Olympus BX-53 light microscope (Olympus, Tokyo, Japan). Quantification of the callose depositions was performed using ImageJ software. Three biological replicates, each with three technical repeats, were conducted for each experiment.

### Hydrogen peroxide and superoxide anion staining

Cut 4-week-old Arabidopsis leaves and place them in a petri dishes containing 1 μM peptide or water. Allow the leaves to incubate in their respective environments for a duration of 1.5 hours. And then transferred to 50 mL of 1% DAB (diaminobezidin) staining solution (1 mg/mL DAB, 5% 200 mol/L Na_2_HPO_4_) or 0.5 mg/mL NBT (tetranitroblue tetrazolium chloride) staining solution, and incubated for 8 h in dark, followed by washing with eluent (ethanol: glycerol: acetic acid = 3:1:1) for 15 min at 95°C. Leaves were observed by Olympus BX-53 light microscope (Olympus, Tokyo, Japan). Three biological replicates, each with three technical repeats, were conducted for each experiment.

### MAPK assay

Fifty 10-days-old seedlings were immersed in sterile water overnight on 1/2 MS solid medium and transferred into a 6-well plate. Peptides were then added to a final concentration of 1 μM for 15 minutes induction. Next, the seedlings were snap frozen in liquid nitrogen and ground to a fine powder, from which total protein was extracted by suspension in protein extract buffer (Tris-HCl (pH 6.8), glycerol 25% (V/V), SDS 2% (V/V), bromophenol blue 0.001% (W/V), mercaptoethanol 5% (V/V)). An anti-phospho p44/p42 MAPK antibody (Huabio, China) was used to detect active MPK6 and MPK3 via immunoblotting. Ponceau staining indicates equal loading of total proteins. Three biological replicates, each with three technical repeats, were conducted for each experiment.

### Stomata observation

Four-week-old Arabidopsis leaves were treated with or without 1 µM small peptide for 4 h. Then, the adaxial epidermal leaf surface was affixed to Scotch transparent adhesive tape with the abaxial side facing upward. Subsequently, another strip of the tape was firmly topped on the abaxial surface of the affixed leaf. The upper tape was then gently pulled away from the lower tape, peeling away the abaxial epidermal cell layer attached to the lower tape. Leaf stomata were observed by Olympus BX-53 light microscope (Olympus, Tokyo, Japan). We used ImageJ software for measuring the widths of stomata. Three biological replicates, each with three technical repeats, were conducted for each experiment.

### Pathogen inoculation assay

The pathogen inoculation methods for *Botrytis cinerea* and *Sclerotinia sclerotiorum* inoculation was based on the methods of to Wang et al. (2015). The strain was cultivated on potato dextrose agar (PDA) for 15 days at a temperature of 23°C, with a 12 h photoperiod. Following that, 4-week-old Arabidopsis leaves were sprayed with a 100 mL solution containing 1 μM of peptides. The plants were left overnight. The leaves were then carefully cut and placed in petri dishes with their petioles embedded in moist cotton. Evenly growing fungal agar blocks (*Botrytis cinerea* and *Sclerotinia sclerotiorum*) with a diameter of 2 mm on the leaves, and photographs were taken after 4 days co-culture in an artificial climate chamber (12 h of light (140 μmol·m^-2^·s^-1^), 22°C, 60% humidity). The ratio of necrotic area to total leaf area on leaves infected by the pathogens was quantifed by ImageJ software. Three biological replicates, each with three technical repeats, were conducted for each experiment.

### RNA-seq

Ten-day-old seedlings grown on 1/2 MS solid medium were transferred to water for overnight-recovery and then treated with 1 μM peptide for 1 h. They were immediately frozen in liquid nitrogen and stored at -80°C for RNA extraction. Total RNA was extracted from frozen Arabidopsis 10-day-old seedlings using an RNAprep Pure Plant Plus Kit (TIANGEN, Beijing, China) according to its instruction manual. RNA-seq and bioinformatics analyses were executed by Personal Biotechnology Cp. Ltd. (Shanghai, China). The sequencing libraries of samples were constructed by a TruSeq RNA Sample Preparation Kit (Illumina, SanDiego, CA, USA), and the libraries were sequenced on the Illumina Hiseq X platform. The reference genome database was extracted from TAIR Database (https://www.arabidopsis.org/index.jsp), and gene annotations were acquired from the genome. The index of the reference genome was built with Bowtie2 (2.2.6), and the clean reads were mapped to the reference genome using HISAT (2.0.5). HTSeq (0.9.1) was applied to the expression and quantification levels of genes to calculate fragments per kilobase per million fragments (FPKM). DEG were identified by DESeq (1.20.0) with screening conditions as follows: expression difference multiple |log2FoldChange| > 1 and significant *P*-value < 0.05. Subsequently, clustering analysis of all differential genes was performed using the “Pheatmap” library in R statistical package (1.0.8). Blast2GO was used for analysis of Gene Ontology (GO) annotation (Conesa et al., 2005), KAAS was used for the KEGG annotations (Moriya et al., 2007). Each point had three biological replicates for RNA-seq analysis.

### Quantitative RT-PCR analysis

RNA-seq samples were used for quantitative RT-PCR validation. Total RNA was extracted from plant tissues by the FastPure Plant Total RNA Isolation Kit (Vazyme, Nanjing, China) following the manufacturer’s protocol. One μg of total RNA was subjected to reverse transcription using the HiScript III 1st Strand cDNA Synthesis Kit (Vazyme, Nanjing, China). The resulting cDNA was amplified using the Taq Pro Universal SYBR qPCR Master Mix (Vazyme, Nanjing, China) and gene-specific primers (**Supplementary Table S1**). AtActin2 was used as the reference sequence. Three biological replicates, each with three technical repeats, were conducted for each experiment.

### Bioinformatics analysis

NCBI BLAST+ v2.13 was used for homology search and HMMER 3.0 were used for iterative comparison (Mistry et al., 2013). Signal peptide prediction was performed by SignalP 5.0 (Almagro et al., 2019). Phylogenetic analysis on the PIP sequence was performed by MEGA 6 (Tamura et al., 2013). Motif prediction was performed by MEME (Bailey et al., 2015). Predicted cis-acting elements was conducted by PlantCARE (Lescot, 2002). Evolution pressure analysis was conducted by SELECTION (Stern et al., 2007). Gene recombination analysis was performed using RDP5 (Martin et al., 2020). Notung v2.9.15 was used to infer gene loss and duplication events (Stolzer et al., 2012). Amino acid conservation analysis was conducted by Weblogo (Crooks et al., 2004). Gene fragment duplication and circos plots were performed using TBtools (Chen et al., 2020b). Molecular docking calculations were performed using HPEPDOCK (Zhou et al., 2018) and related analyses were conducted using PDBePISA (https://www.ebi.ac.uk/msd-srv/prot_int/pistart.html). Gene coexpression analysis was performed using Arabidopsis RNA-seq Database (Zhang et al., 2020). Protein interaction networks were made using the STRING database (Szklarczyk et al., 2020) and Cytoscape (Shannon et al., 2003). The expression of AtprePIP1 and AtprePIP2 in roots were obtained from BAR Expression (Fucile et al., 2011). The expression of AtprePIP1 and AtprePIP2 under fungal infection were obtained from GEO database (GSM2850645, GSM2850646, GSM2850647, GSM1618754, GSM1618755, GSM1618756).

### Accession numbers

Transcriptome data raw reads are available at the NCBI BioProject database (http://www.ncbi.nlm.nih.gov/bioproject) under Bioproject ID: PRJNA924544.

### Statistical analysis

Data obtained were subjected to statistical analysis using analysis of variance (ANOVA) procedures to test the significant effect of all the variables investigated, using IBM SPSS Statistics version 26. Means were separated using Duncan Multiple Range Test (DMRT) as the test of significance at *p* < 0.05. TBtools, Excel 2019 and Origin 2019b were used for mapping.

## Results

### Identification and classification of PIP families in different species

**Figure 1:**
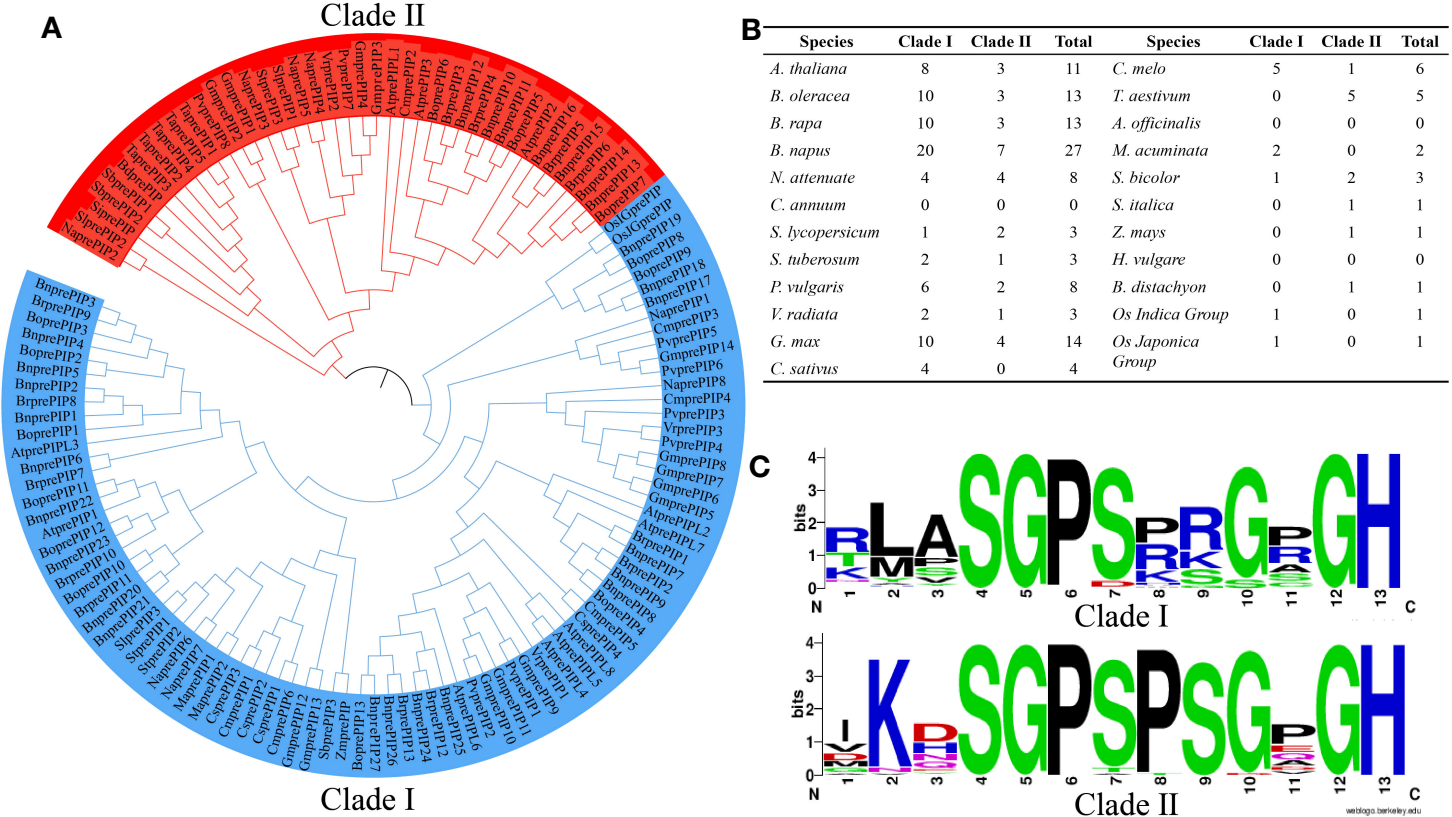
Distribution of 128 PIP family members in 23 plant species. **(A)** Evolutionary tree branches of 128 PIP family members. **(B)** The number of PIP family members in each Clade. **(C)** Degree of amino acid conservation in each Clade.

Twenty-three common and representative plant (13 dicotyledons and 10 monocotyledons species from 7 families) were selected from the Ensemble plants database (http://plants.ensembl.org/index.html) (Yates et al., 2021). Using Arabidopsis PIPs as template (**Supplementary Figure S1**), a total of 128 PrePIPs were identified by iterative comparison with Hmmer and Blast+, and signal peptide search with SignalP 5.0 (**Supplementary Table S2**). Phylogenetic analysis was performed by Muscle using MEGA 6.0, which distinctly classified the PrePIPs proteins into two clades, Clade I and Clade II. Among the two clades, Clade I had more PrePIPs (67.97%) than that of Clade II (**Figure 1A**) among most species (**Figure 1B**). Based on the Weblogo analysis of the two Clades, it was found that the conserved sequences of the two Clades were basically similar, with Clade I has a total of 5 conserved amino acid positions and Clade II has 6 (**Figure 1C**).

### The structure and evolution of PIP family

**Figure 2:**
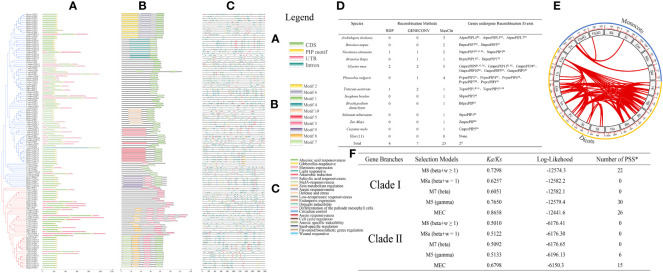
The structure and evolution of 128 PIP family members. **(A)** Gene structure of 128 PIP family members. **(B)** Distribution of motifs in 128 PIP family members. **(C)** Distribution of cis-acting elements in 128 PIP family members. **(D)** Genetic recombination events occurring within the 128 PIP family members in 23 plant species. **(E)** Fragment replication of 128 PIP family members in 23 plant species. **(F)** Evolution pressure analysis of PIP family members. PSS: Positive selection site.

By analyzing the gene structure and motifs of PrePIP sequences, Clade I and Clade II were found to be highly similar in gene structure (**Figure 2A**). The motifs in proteins exhibit some variation, and Clade II’s pattern structure is thoroughly conserved, with motif 3 either occurring independently or in association with motif 7 (as illustrated in **Figures 2B**, **S2**). When cis-acting elements were analyzed, no difference was found in cis-acting element species between Clade I and Clade II, but there was a difference in the number, perhaps it was caused by the number of PIP family members (**Figures 2C**, **S3**). It is noteworthy that the ABA response element didn’t differ significantly between Clade I and Clade II (**Figure S3**). We then applied three methods to confirm that PIPs do undergo genetic recombination events among species, but the number is relatively small (**Figure 2D**). We further analyzed arrangement of orthologs in different species, and found that this phenomenon exists in PIPs and is predominant in dicotyledons (**Figure 2E**). It is interesting to note that monocotyledons and dicots gene duplication events independently. We selected five mathematical models for evolutionary direction analysis, and found that the *Ka/Ks* results of all five mathematical models were less than 1 (**Figure 2F**), indicating that the evolution direction of PrePIPs is purification selection. In addition, purification selection occurs in all amino acids in the PIP region (**Figure S4**). Meanwhile, gene duplication analysis results indicate that gene duplications (60) and gene losses (94) may have occurred in PIP gene family during evolution (**Figure S5**).

Weblogo analysis of Clade I and Clade II revealed that Arabidopsis AtPIP1 and AtPIP2 are the closest natural sequences of Clade I and Clade II, respectively (**Figure 1C**, **Supplementary Figures S1**, **S6A**). And it has been shown that PIP belongs to a peptide rich in SGP motif, which undergoes hydroxylation in its SGP (Hou et al., 2014). Interestingly, one SGP is found in AtPIP1, while two SGPs are found in AtPIP2, which maybe undergo hydroxylation twice. We found higher expression of AtprePIP1 than AtprePIP2 in roots in the BAR database and higher expression of AtprePIP2 than AtprePIP1 under fungal infestation by the GEO database (**Supplementary Figure S6B**).

### AtPIP1 inhibits root growth more significantly than AtPIP2 in Arabidopsis

**Figure 3:**
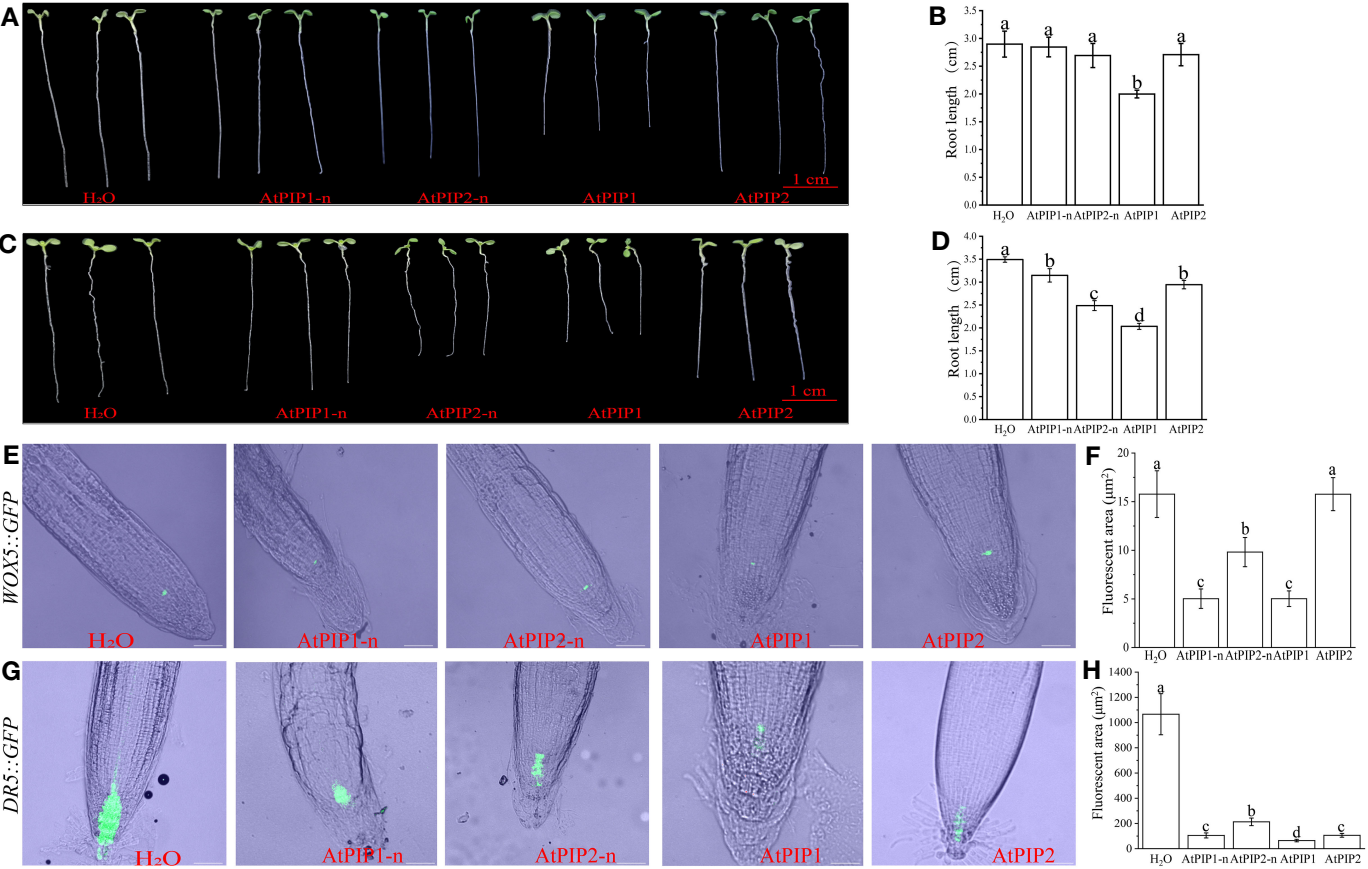
Root growth is inhibited by AtPIPs (AtPIP1, AtPIP1-n, AtPIP2, and AtPIP2-n). **(A)** Effect on root growth upon 100 nM AtPIPs treatment. **(B)** Statistic analysis of primary root length upon 100 nM AtPIPs treatment. Error bars represent the SE of the mean (n>5). Statistically significant differences (*p*<0.01) are indicated by different letters. **(C)** Effect on root growth upon 1 μM AtPIPs treatment. **(D)** Statistic analysis of primary root length upon 1 μM AtPIPs treatment. Error bars represent the SE of the mean (n>5). Statistically significant differences (*p*<0.01) are indicated by different letters. **(E)** Effect of 1 μM AtPIPs on Arabidopsis seedlings expressing *WOX5::GFP*. **(F)** Statistic analysis of 1 μM AtPIPs on Arabidopsis seedlings expressing *WOX5::GFP*. **(G)** Effect of 1 μM AtPIPs on Arabidopsis seedlings expressing *DR5::GFP*. **(H)** Statistic analysis of 1 μM AtPIPs on Arabidopsis seedlings expressing *DR5::GFP*. Number>5. Scale bars = 50 μm.

Previous studies showed that both AtPIP1 and AtPIP2 inhibited Arabidopsis root growth, however differences in activity between one hydroxylated AtPIP1 and two hydroxylated AtPIP2 were not noticed (Hou et al., 2014). Therefore, we performed detailed root growth inhibition assay using AtPIP1 and AtPIP2 with or without hydroxylation modification at 100 nM or 1 μM. Specifically, the peptides used were AtPIP1-n (RLASGPSPRGRGH), AtPIP2-n (VKHSGPSPSGPGH), AtPIP1 (RLASGPHpySPRGRGH), and AtPIP2 (VKHSGPHpySPSGPHpyGH). The results revealed that, comparing with AtPIP2, AtPIP1 significantly inhibited root growth at either 100 nM or 1 μM, and the inhibition activity was largely influenced by the proline hydroxylation modification (**Figures 3A–D**). To understand the basis of growth regulation, we explored the gene expression of certain genes involved in root proliferation. The WOX5 protein (wuschel-associated homeobox 5) is specifically expressed in the quiescent center (QC) cells of root tip meristem and maintains surrounding stem cell activity (Liu et al., 2014). We treated Arabidopsis seedlings carrying *WOX5::GFP* with the above peptides and found that compared to the control, the expression of *WOX5::GFP* in AtPIP1-n and AtPIP1 treatment was significantly lower, and there was no difference between AtPIP1-n and AtPIP1 treatments. Under AtPIP2 treatment, the expression of *WOX5::GFP* remained unchanged, while after AtPIP2-n treatment, the expression of *WOX::GFP* was significantly reduced, but still higher than AtPIP1-n and AtPIP1 treatment (**Figures 3E, F**). DR5 is a auxin reporter gene in Arabidopsis roots (Benková et al., 2003), and we observed a significant decrease in the expression of *DR5::GFP* gene in Arabidopsis seedlings treated with AtPIPs compared to the control, with AtPIP1 treatment being the most significant (**Figures 3G, H**). The above results indicate that although both AtPIP1 and AtPIP2 affect root development, AtPIP1 inhibits root growth more significantly than AtPIP2 in Arabidopsis.

### AtPIP2 induces stronger PTI than AtPIP1 in Arabidopsis

**Figure 4:**
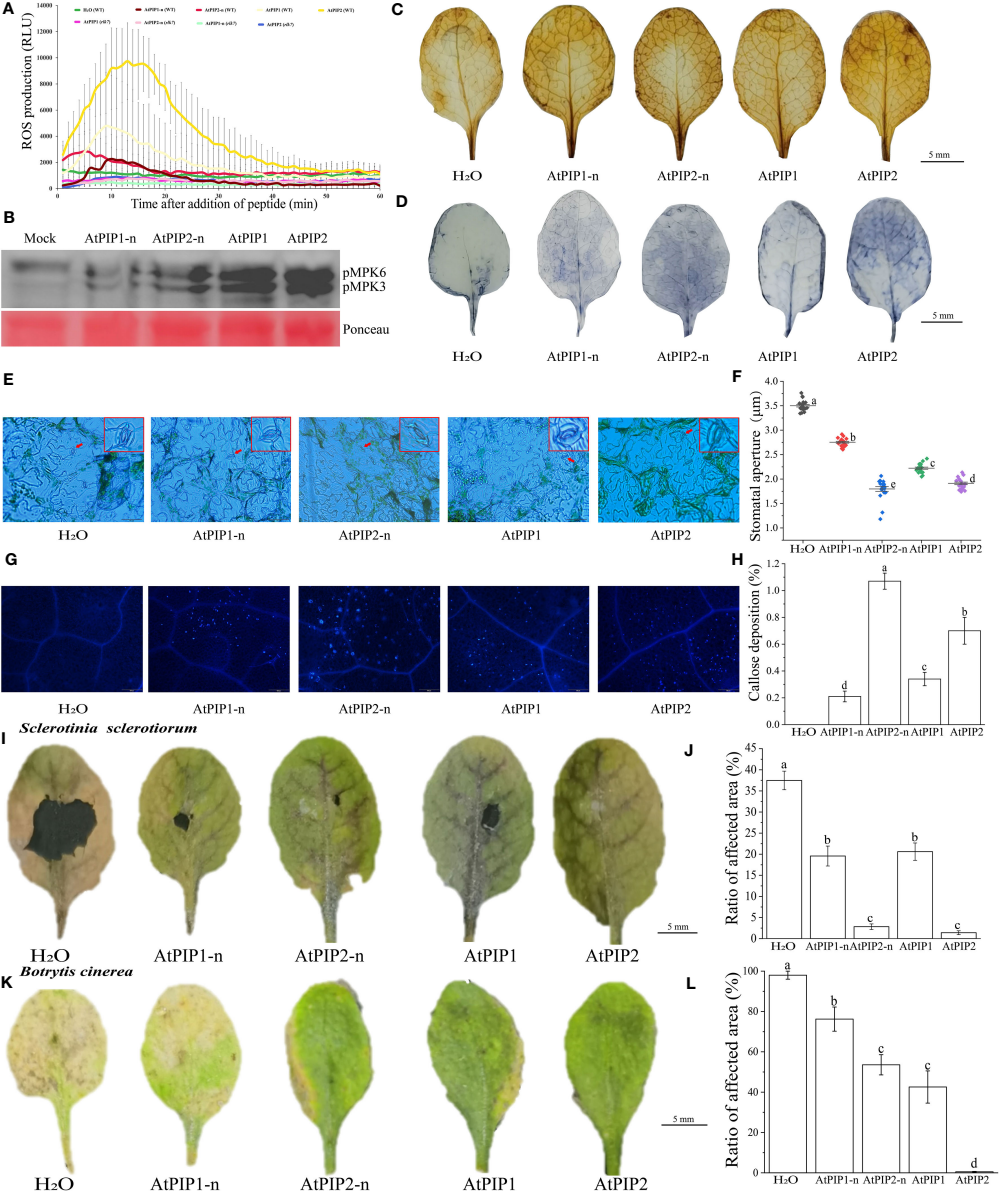
AtPIPs (AtPIP1, AtPIP1-n, AtPIP2, and AtPIP2-n) induce plant immune response in Arabidopsis. **(A)** AtPIPs induce a ROS burst. Leaf discs from four-week-old soil-grown plants were treated with or without 100 nM AtPIPs, and ROS production was measured as relative light units (RLU) by a luminometer over 60 min. Error bars represent the SE of the mean (n>3). **(B)** MAPK activation by AtPIPs in Arabidopsis seedlings. Ten-day old seedlings were exposed to 1 μM peptide for 15 min. Western blot analysis was performed with the phospho-p44/42 MAPK antibody. Three replicates were performed with similar results. **(C)** DAB staining for hydrogen peroxide in plant leaves under AtPIPs treatment. **(D)** NBT staining for superoxide in Arabidopsis leaves under AtPIPs treatment. **(E)** Fluorescence microscopy imaging and **(F)** quantification of stomatal closure induced by AtPIPs. **(G)** Fluorescence microscopy imaging and **(H)** quantification of AtPIPs-induced callose deposition in plant leaves. Error bars represent the SE of the mean (n>10). **(I)** *Botrytis cinerea* symptoms on Arabidopsis leaves under AtPIPs treatment. **(J)** Measurements of lesion areas on Arabidopsis leaves 4 days post inoculation (DPI). Error bars represent the SE of the mean (n>3). **(K)** *Sclerotinia sclerotiorum* symptoms on Arabidopsis leaves under AtPIPs treatment. **(L)** Measurements of lesion areas on Arabidopsis leaves 4 DPI. Error bars represent the SE of the mean (n>3). Statistically significant (*p*<0.01) differences are indicated by different letters.

AtPIPs induce PTI response such as ROS burst, MAPK activation, stomatal closure and callose deposition in Arabidopsis, leading to pathogen resistance (Hou et al., 2014). Through the reactive oxygen burst experiment, we found that the activity of AtPIP2 was higher compared to AtPIP1, and AtPIPs did not respond in the *rlk7* mutant (**Figure 4A**). Analysis of MAPK activation upon peptides treatments, showed that the activity of AtPIP2 is stronger than that of AtPIP1 and both AtPIPs required hydroxylation modification to acquire full activity (**Figure 4B**). DAB and NBT staining showed that although all four treatments caused hydrogen peroxide and superoxide anion accumulation, AtPIP2 was stronger than AtPIP1 (**Figures 4C, D**). Similarly, stomatal closure and callose deposition observations indicated that AtPIP2 had a higher effect than AtPIP1 (**Figures 4E–H**).

Pathogen infection experiments showed that upon *Sclerotinia sclerotiorum* and *Botrytis cinerea* inoculation, AtPIP2 and AtPIP2-n treated leaves were more resistant than AtPIP1 and AtPIP1-n, respectively (**Figures 4I–L**). Therefore, combining the above results, AtPIP2 triggers stronger plant immune responses than AtPIP1.

### Transcriptome changes in Arabidopsis seedlings under AtPIP1 and AtPIP2 treatments

**Figure 5:**
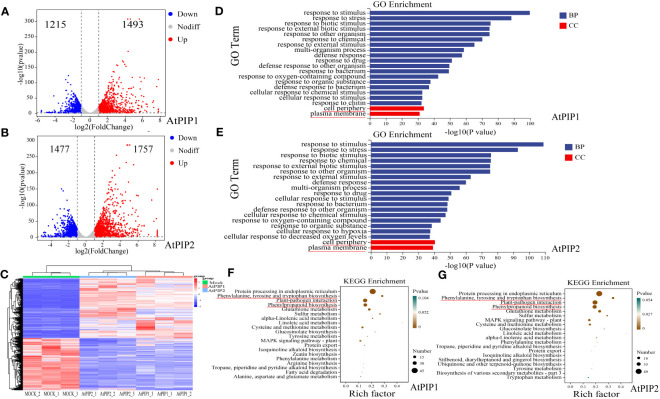
Transcriptome of Arabidopsis seedlings after 1 h of AtPIP1 and AtPIP2 treatment. **(A)** Volcano plot displays differentially regulated genes (DEGs) with fold change (FC) > 2 and *P* < 0.05 at 1 h after AtPIP1 treatment in three biological replicates. **(B)** Volcano plot displays differentially regulated genes (DEGs) with fold change (FC) > 2 and *P* < 0.05 at 1 h after AtPIP2 treatment in three biological replicates. **(C)** Hierarchical clustering of DEGs at 1 h after AtPIP1 and AtPIP2 treatment. Expression levels of each gene across different samples are shown as Z–scores scaled by the FPKM (fragments per kilobase of transcript per million mapped reads). **(D)** GO analyses of AtPIP1-upregulated genes. **(E)** GO analyses of AtPIP2–upregulated genes. **(F)** KEGG analyses of AtPIP1-upregulated genes. **(G)** KEGG analyses of AtPIP2-upregulated genes. BP, biological process. CC, cellular compartment. **(D–G)** The red line represents the noticed terms.

To better elucidate the molecular effects of AtPIP1 and AtPIP2 on growth and resistance to biotic stress in Arabidopsis, we selected AtPIP1 and AtPIP2 for RNA-seq analysis of Arabidopsis seedlings after 1 h of treatment (**Supplementary Figure S7A**), as it is generally believed that 1 h of treatment can represent early responses to such signals at the gene regulatory level (Xoca-Orozco et al., 2017). It was found that AtPIP1 induced up-regulation of 1493 genes and 1215 genes were down-regulated in Arabidopsis seedlings (Log2Fold>1, P-value ≤ 0.05), while AtPIP2 induced the up-regulation of 1757 genes and 1477 genes were down-regulated (Log2Fold>1, P-value ≤ 0.05) (**Figures 5A, B**). The genes were hierarchically clustered on the basis of their differential expression and the statistical significance (**Figure 5C**). Up-regulated genes were then analyzed by Gene Ontology (GO) and the Kyoto Encyclopedia of Genes and Genomes (KEGG), respectively. GO analyses showed that the two enrichment pathways were similar and basically enriched in biological processes (BP) such as stress response and organism process (**Figures 5D, E**), indicating their functional roles in growth and stress resistance. Enriched cellular components (CC) are mainly membrane-associated, which reflects the model of AtPIP’s action is via its receptor and signaling complex on the membrane. KEGG analysis showed that both AtPIPs were also involved in essentially the same synthetic pathways, but AtPIP2 was found to be more involved in the phenylpropanoid pathway and plant-pathogens interactions (88) compared to AtPIP1 (71). These results further elaborated why AtPIP2 induced higher pathogens resistance (**Figures 5F, G**).

**Figure 6:**
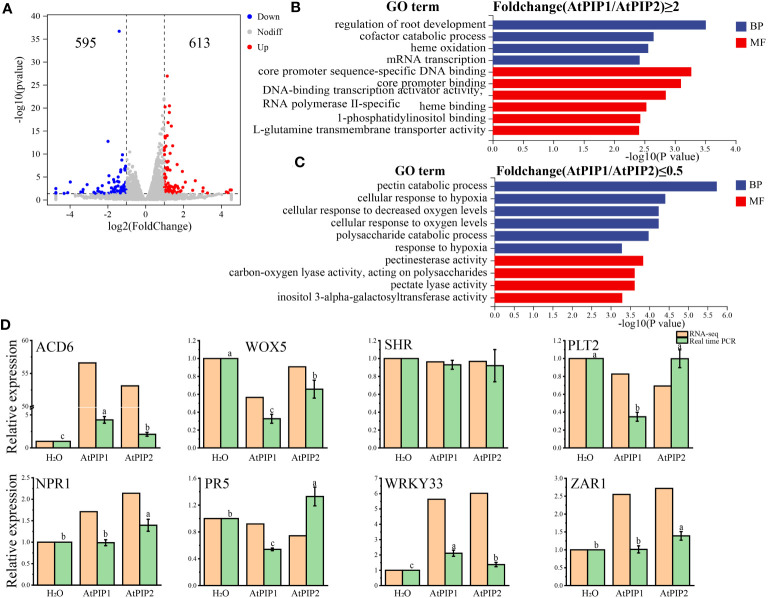
Comparative analysis of transcriptome DEGs after 1 h of AtPIP1 and AtPIP2 treatment. **(A)** Volcano plot displays differentially regulated genes (DEGs) with fold change (FC)>2 and *P*<0.05 between treatment with AtPIP1 and AtPIP2. **(B)** GO analyses of AtPIP1–upregulated (Fold change (AtPIP1/AtPIP2)≥2) genes. **(C)** GO analyses of AtPIP1–downregulated (Fold change (AtPIP1/AtPIP2)≤0.5) genes. **(D)** RT-qPCR validation (green) of RNA-seq data (orange) using eight genes. Expression levels of these genes in WT plants at 1 h after mock treatment were normalized as 1. Error bars represent the SE of the mean (n=3). Statistically significant (*p*<0.01) differences indicated by different letters. BP, biological process. CC, cellular compartment.

Next, we compared transcriptome differences between AtPIP1 and AtPIP2 treatments. Compared to AtPIP2, AtPIP1 yielded 613 up-regulated genes, and down-regulated genes were 595 (Log2Fold>1, P-value ≤ 0.05) (**Figure 6A**). Through GO enrichment analysis, it was found that the significantly up-regulated genes are mainly enriched in the regulation of root development (**Figure 6B**), which is consistent with our previous experimental results. The Gene Ontology categories in down-regulated genes were mainly enriched in pectin catabolism and response to oxygen (**Figure 6C**), which also contributes to the enhancement of plant immunity. We selected specific genes related to growth and defense mechanisms to access that how they are activated in both AtPIP1 and AtPIP2. To validate the above RNA-seq data, the expression of eight genes was further confirmed by quantitative reverse transcription PCR (qRT-PCR) (**Figure 6D**).

### Co-expressed genes of AtPIP1 and AtPIP2 are enriched to the immune pathway

**Figure 7:**
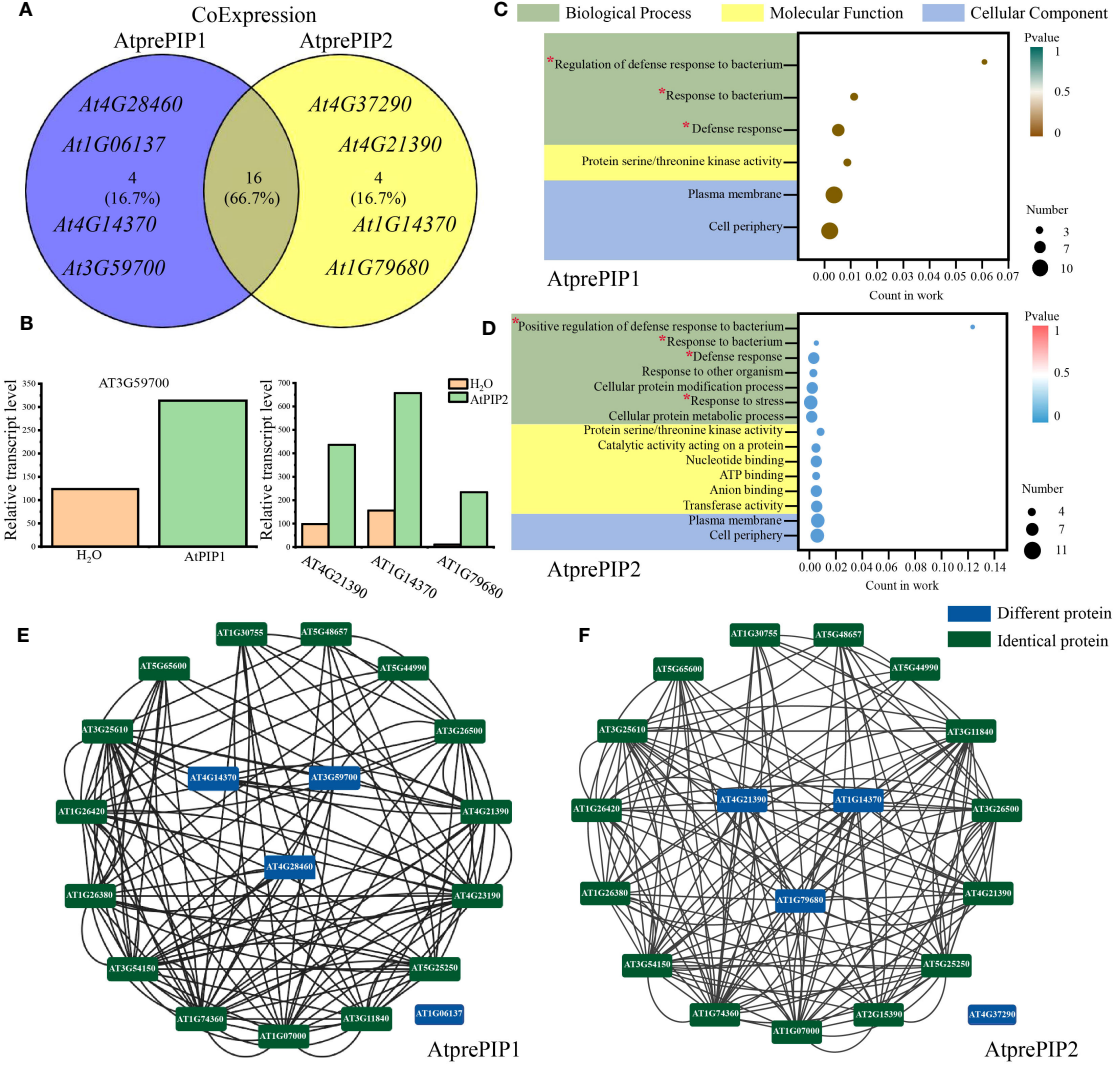
Comparative analysis of co-expressed genes of AtprePIP1 and AtprePIP2. **(A)** Identification of co-expressed genes of AtprePIP1 and AtprePIP2 using Arabidopsis RNA-seq database. **(B)** Expression of differentially co-expressed genes in Arabidopsis seedlings after 1 h of AtPIP1 and AtPIP2 treatment. **(C)** GO enrichment analysis of the subnetwork in AtprePIP1 co-expressed genes. **(D)** GO enrichment analysis of the subnetwork in AtprePIP2 co-expressed genes. The red * represents the noticed terms. Protein-protein interaction network of AtprePIP1 **(E)** or AtprePIP2 **(F)** co-expressed genes. The black lines represent protein-protein interaction. The green frame represents the identical protein, while the blue frame represents the differential proteins co-expressed with AtprePIP1 or AtprePIP2.

We performed Co-expression analysis (Zhang et al., 2020) using Arabidopsis RNA-seq Database (http://ipf.sustech.edu.cn/pub/athrdb/) and selected TOP20 co-expressed genes (**Supplementary Table S3**). We observed that AtprePIP1 and AtprePIP2 were identical in expression patterns and there were four different genes (**Figure 7A**). Combined with the transcriptome, one immune related gene *AT3G59700* (lectin-receptor kinase, HLECPRK) was found to be up-regulated by AtPIP1, while three genes *AT4G21390* (S-locus lectin protein kinase family protein, B120), *AT1G14370* (protein kinase 2A, APK2A) and *AT1G79680* (WALL ASSOCIATED KINASE (WAK)-LIKE 10, WAKL10) were up-regulated by AtPIP2 (**Figure 7B**). According to GO terms and gene interaction numbers, we found that the interaction between AtPIP2 and its co expressed genes (19) is more enriched in the immune pathway than AtPIP1 (13) (**Figures 7C, D**). Prediction of protein interactions (black line in figure) by PPI in String (https://cn.string-db.org/) showed that AtPIP2 co-expressed genes had more protein interactions than AtPIP1 (**Figures 7E, F**; **Supplementary Table S4**). It is unclear why AtPIP2, different from PIP1, does not interact with any members in the network. We also noticed that an identical gene *AT2G25297* is not included in the database and thus missed in the results. In consistent with previous results, the above analysis also indicates that AtPIP2 potentially have a stronger pathogens resistance effect over AtPIP1.

### Molecular docking of AtPIP1 and AtPIP2 with RLK7

**Figure 8:**
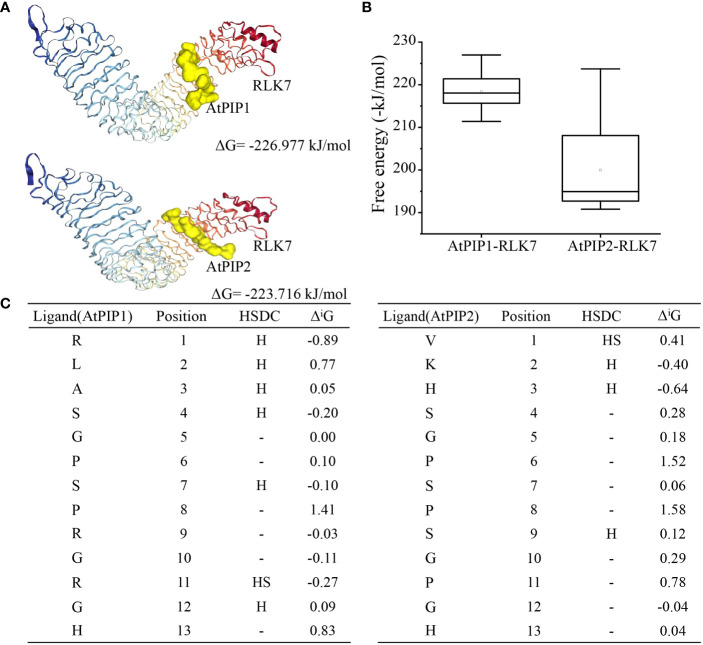
Molecular docking model of the AtPIP1 and AtPIP2 with RLK7. **(A)** The most stable structure model formed by binding of AtPIP1 and AtPIP2 to RLK7. **(B)** Analysis of the 10 lowest energies formed by binding of AtPIP1 and AtPIP2 to RLK7. **(C)** Free bonds and energies resulting from binding of AtPIP1 and AtPIP2 to RLK7. HSDC stands for hydrogen, salt bridge, disulphide bond and covalent link, respectively.

Recognition and binding to the receptors are essential for the biological function of small secreted peptides (Song et al., 2016), such as perception of AtPIP1 and AtPIP2 by RLK7 (Hou et al., 2014). To explore the binding of AtPIP1 and AtPIP2 to the receptor, we performed semi-flexible docking by HPEPDOCK2.0 (http://huanglab.phys.hust.edu.cn/hpepdock/) and acquired 10 most stable structures (**Supplementary Figure S8A**). Gibbs free energy is ΔG = -226.977 kJ/mol for the most stable structure of AtPIP1 binding to RLK7, while Gibbs free energy is ΔG = -223.716 kJ/mol for the most stable structure of AtPIP2 binding to RLK7 (**Figure 8A**). The stability of a complex increases as the binding energy between the ligand and receptor decreases (Que et al., 2021). We found that the free energy of AtPIP1 binding to RLK7 is lower than that of AtPIP2, i.e., binding of AtPIP1 to RLK7 is more stable (**Figures 8A, B**). Next, we selected the most stable structures of AtPIP1 and AtPIP2 with RLK7 for the analysis of relevant parameters. When bond to RLK7, both the atomic and residue interface of AtPIP2 are higher than those of AtPIP1, while the surface is the same. Similarly, the solvent-accessible interface of PIP2 is larger than AtPIP1, while gain on complex formation and average gain are both less than AtPIP1 (**Supplementary Figures S8B, C**). Therefore, AtPIP2 would have a larger contact area and lower solvation energy when bound to RLK7. Interestingly, while AtPIP1 generates six hydrogen bonds and one ionic bond upon binding, AtPIP2 generates four hydrogen bonds and one ionic bond (**Figure 8C**). Structural analysis revealed that a hydrogen bond is generated at the Ser position of the AtPIP1 SGP motif, while the second SGP motif but not the first SGP motif of AtPIP2 generates a hydrogen bond. It was also found that AtPIP1 and AtPIP2 have different ion binding positions when binding to RLK7, with AtPIP1’s ion bond binding at R11 and AtPIP2’s ion bond binding at V1. Interestingly, R11 of AtPIP1 corresponds to P11 in the second SGP motif of AtPIP2. Together, according to computer simulation results, the SGP motif has a great influence on the bond energy generated by PIP-RLK7 binding.

## Discussion

### Functional divergence of PIPs

**Figure 9:**
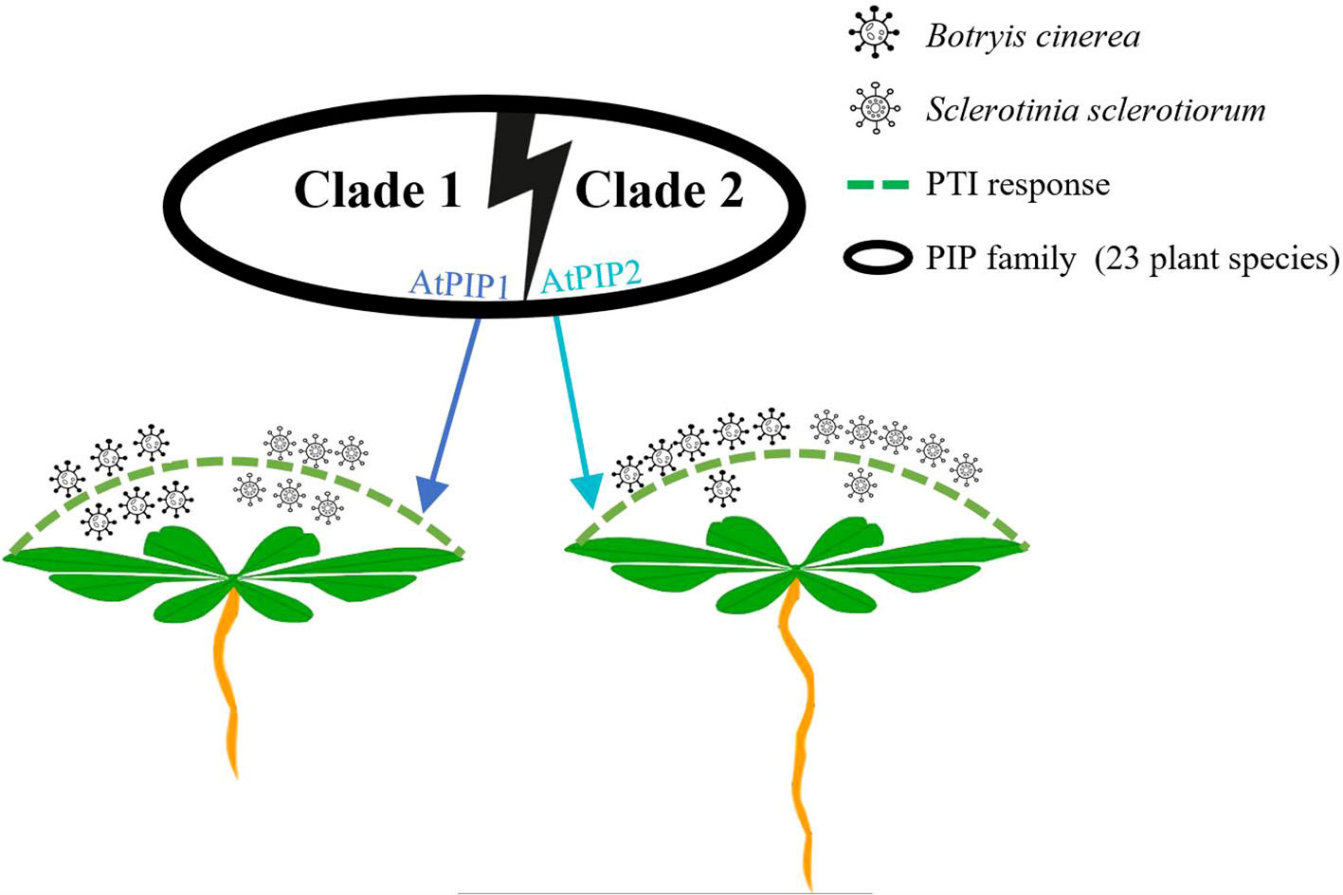
Schematic model illustrating functional divergence of AtPIP1 and AtPIP2 in Arabidopsis.

In this study, the two main representative members of the PIP peptide family, AtPIP1 and AtPIP2, were found to share many similarities in amino acid composition, triggering PTI responses and inhibiting root growth in Arabidopsis (**Figures 3**, **4**, **6**). This result is similar to Huang et al. (2023), that peptide elicitors often not only induce immune response but also inhibit root growth, such as flg22, pep1 and nlp20. However, more detailed analysis indicated that AtPIP1 had more impact on root growth inhibition, while AtPIP2’s induced a stronger immune response (**Figure 9**). Therefore, immune and inhibitory root length are not fully coupled for PIPs. Activation of immunity by exogenous signals leading to autoimmunity has long been associated with decreased plant growth, known as the growth-defense trade-off. Recent studies have demonstrated that growth and defense can be uncoupled for endogenous peptides (Berry and Argueso, 2022). For example, Pep peptides trigger immune response, root growth inhibition and root hair formation through cell surface receptors PEPR1 and PEPR2 in Arabidopsis. Analysis of Arabidopsis plants that specifically express PEPR2 in root hair cells revealed uncoupling of root growth inhibition and root hair formation with defense activation (Okada et al., 2021). Similarly, RGF (root growth factor) is a classic plant endogenous peptide that promotes root growth, among which RGF7 has been revealed to have immune functions (Wang et al., 2021). RALF23 peptide suppresses immunity and inhibits root growth, whereas RALF17 peptide enhances immunity. RALF17 and RALF23 are categorized as members of two separate clades based on the S1P clearance site and are devoid of a proposal region (Stegmann et al., 2017). Similarly, CLE9/10 from the CLE family regulates xylem development, while CLE25/45 regulates bast development (Willoughby and Nimchuk, 2021). CLE9/10 and CLE15/45 are also classified as members of different clades in Arabidopsis (Zhang et al., 2022). Therefore, members of different clades can exhibit different functions, and highlighting the separation between growth and immunity.

Functional divergence is a known existing phenomenon in the plant kingdom, and after duplication, a gene duplication often accumulates mutations that may lead to functional divergence (Liu et al., 2018). A duplication of paralog pairing from fragment replication may often evolve through different mechanisms, such as neo functionalization, subfunctionalization, and nonfunctionalization (Xu et al., 2020). The diverse functions of different members within these peptide family are probably due to recognition of individual peptide by different receptors at specific tissues or cell types. Interestingly, AtPIP1 and AtPIP2 require the same receptor, RLK7, to induce immune response (Hou et al., 2014) and the peptides were applied exogenously to the plant in this study. Therefore, the underling mechanism for function divergence of PIP family is likely different from the above peptide families.

The peptide sequence of AtPIP1 and AtPIP2 differs in five amino acids (**Supplementary Figure S6**). Characteristic peptide sequences have been reported to be critical for receptor recognition. For example, deletion of the last amino acid Asn^23^ of AtPep1 significantly compromised AtPep1’s interaction with PEPR1LRR (Tang et al., 2015), and the Arg-x-Gly-Gly (RxGG) motif is responsible for specific recognition of the sulfate group of RGF1 by RGFR1 (Song et al., 2016). The molecular docking results revealed that AtPIP1-RLK7 and AtPIP2-RLK7 complexes have different contact areas, solvation energies, and bond types and quantities (**Figure 8**), which may cause them to recruit relevant co-receptors and other signaling components with different energy, leading to differences in preferred composition of final AtPIP1-RLK7 and AtPIP2-RLK7 signaling complexes. As the final signaling output is a combined effect of all complexes with differences in components such as distinct members of SERKs and RLCKs, such difference may eventually lead to varied plant response generated by different peptides from the same peptide family.

Functional divergence may also occur at transcriptional level. By analyzing cis-acting elements, we found that Clade I generally possess a greater number of cis-acting elements compared to Clade II. However, it is noteworthy that the opposite trend is observed for ABA (abscisic acid) responsive elements, with Clade II exhibiting a higher number of cis-acting elements comparing to Clade I. ABA serves as a crucial plant hormone that regulates the adaptive response of plants to various environmental stress conditions (De-Zelicourt et al., 2016). Therefore, gene regulation by genetic studies should be analyzed to fully understand the detailed function of individual PIP family members in the future.

### Similarities and differences of AtPIP1 and AtPIP2 at transcription level

Through transcriptome analysis, we found that the GO and KEGG enrichment pathways were similar for both AtPIP1 and AtPIP2 (**Figure 5**). Both peptides were enriched in pathways related to stimulus response and phenylalanine, which can help plants resist pathogens (Zhou and Zhang, 2020; Shen et al., 2022). When comparing the transcriptome of AtPIP1 and AtPIP2 directly, the AtPIP1 up-regulated genes were mainly related to auxin and root growth, with GO pathways enriched in regulating root development, indicating that AtPIP1 has a stronger effect on regulating root growth than AtPIP2. Compared with AtPIP2, the lower expressed genes of AtPIP1 were mainly enriched in pectin catabolic and response to hypoxia and oxygen (**Figure 6C**). Pectin, as one of the main components of polysaccharides in the primary cell wall structure, plays an important regulatory role in the process of plant immune resistance (Athanas et al., 2022). As one of the mediators of plant immunity, the activity and content of oxygen are closely related to plant immunity (Hsu et al., 2013). The WOX5 protein (wuschel-associated homeobox 5) is specifically expressed in the quiescent center (QC) cells of the root tip meristem and maintains the surrounding stem cell activity (Liu et al., 2014). As a positive regulator of root growth, the expression of WOX5 is higher after AtPIP2 treatment compared to AtPIP1 treatment (**Figures 3E**, **6D**). The differences in expression of above genes may be one of the reasons why AtPIP1 inhibitors root length more significantly than AtPIP2 and AtPIP2 has a stronger immune effect than AtPIP1.

Gene co-expression analysis revealed four differentially expressed genes co-expressed with AtPIP1 and AtPIP2. Among these, the AtPIP1-induced highly expressed gene was *AT3G59700*, which was annotated as L-type lectin receptor kinase-V.5 (LecRK-V.5), a negative regulator of stomatal immunity (Desclos-Theveniau et al., 2012). In contrast, there were three highly expressed genes in AtPIP2 treatment, *AT4G21390*, *AT1G14370*, and *AT1G79680*. *AT4G21390* belongs to the lectin protein kinase family, which helps Arabidopsis resist pathogen infestation (Cole and Diener, 2013). *AT1G14370* is known as PBL2, which activates the ZAR1-RKS1 complex to trigger plant immunity (Zhang et al., 2010; Wang et al., 2019). *AT1G79680* is annotated to be WAKL10, which can co-express with well-characterized pathogen defense-related genes and is an early responsive gene to a range of pathogens and their elicitors (Meier et al., 2010). Therefore, AtPIP2 co-expressed genes in the GO analysis are more enriched in the pathogen resistance pathway which may also suggest that AtPIP2 is more effective in pathogen resistance than AtPIP1.

### Function and modification of SGP motif in AtPIPs

In the root growth inhibition assay, AtPIP1 showed strongest effect, while vice versa for the AtPIP1-n. Similarly, the pathogen resistance effect of AtPIP2 is better than that of AtPIP2-n (**Figures 4J, L**). These result are similar to previous studies, where P(Pro) hydroxylation modification of the SGP enhanced the function of PIPs (Hou et al., 2014). Although AtPIP1 and AtPIP2 are members of subfamilies with one and two SGP motif. At first, we thought that the double SGP motifs might be clustered together, but we found that 2 out of 80 Clade I members also contains double SGPs. This may be due to the fact that the signal peptide and variable region sequences in front of the peptide were considered for sequence similarity clustering.

Since both hydrogen and ionic bonding changes are related to the SGP motif, we speculate that the functional divergence of PIP family members is partially related with this motif (**Figure 8C**). Many small peptides contain SGP motifs, such as PIP (Hou et al., 2014), CLE (Fletcher et al., 1999), and IDA (Vie et al., 2015) etc. Most members of the IDA family have SGP, a highly conserved motif that undergoes hydroxylation (Matsubayashi, 2014). The SGP motif of CLV3 members in the CLE family is hydroxylated, and glycosylation also occurs after hydroxylation (Ohyama et al., 2009). The critical importance of arabinosylation of CLV3 class peptides is highlighted by the identification of the *Lotus japonicus* CLE-RS2 glycopeptide, which is involved in autoregulation of nodulation (Okamoto et al., 2013). The mechanism behind the enhanced growth and immune effects of P hydroxylation in SGP motif remains to be investigated. In our study, application of small peptides is *in vitro*, as modifications in plants may occur under specific conditions. Therefore, *in vivo* studies on PIP small peptides are necessary in the future.

## Conclusion

In summary, 128 PIPs from 23 plant species were identified and classified into 2 clades. This study indicates that AtPIP1 and AtPIP2 are representative sequences of each clade of the PIP family. AtPIP1 effectively inhibits root growth in Arabidopsis, while AtPIP2 can induce immunity more efficiently. These results reveal the functional diversity of the PIP family and provide new insights into the correlation between growth inhibition and immune response. Furthermore, since exogenous application of PIPs based on their specific functions may contribute to pathogen resistance without severe root growth penalty in plants, AtPIP2 can potentially be used for crop protection in the field.

## Data availability statement

The datasets presented in this study can be found in online repositories. The names of the repository/repositories and accession number(s) can be found below: https://www.ncbi.nlm.nih.gov/, PRJNA924544.

## Author contributions

X-SY and YC conceived and designed the experiments. X-SY, F-FL and R-QL performed the biological assays. H-RW and N-UA performed the molecular docking studies and in planta gene expression analysis. X-SY, H-PY, J-BS and YC analyzed the data and finalized the manuscript, which was approved by all the authors.

## Supplementary material

Supplementary Table 1 Primers for qRT-PCR gene expression verification.

Supplementary Table 2 Informations of 128 PIP family members in 23 plant species.

Supplementary Table 3 Top 20 co-expressed genes of AtprePIP1 and AtprePIP2 from Arabidopsis RNA-seq database.

Supplementary Table 4 Protein interactions of AtPIP1 or AtPIP2 co-expressed genes.

Supplementary Figure 1 Gene structure of 11 PIP family members in Arabidopsis.

Supplementary Figure 2 The MEME suite was used to identify conserved motifs of 128 PIP family members.

Supplementary Figure 3 Distribution of cis-acting elements with related functions in Clade I and Clade II.

Supplementary Figure 4 Mutation of amino acid sites in five models were analyzed by SELECTION.

Supplementary Figure 5 Duplications and losses of gene families in different species.

Supplementary Figure 6 Characteristics and precursor gene expression of AtPIP1 and AtPIP2. **(A)** Proportion and dominant PIPs of Clade I and Clade II. **(B)** Expression of AtprePIP1 and AtprePIP2 in Arabidopsis root from BAR database and after infection by *Sclerotinia sclerotiorum* and *Botryis cinerea* from GEO database.

Supplementary Figure 7 Transcriptome analysis after 1 h of AtPIP1 and AtPIP2 treatment. **(A)** Principal Component Analysis (PCA) of Arabidopsis seedlings treated with AtPIP1 and AtPIP2. **(B)** Differences in highly expressed genes after 1 h of AtPIP1 and AtPIP2 treatment.

Supplementary Figure 8 Binding parameters of AtPIP1 and AtPIP2 with the RLK7 obtained from molecular docking. **(A)** The 10 most stable structures when AtPIP1 and AtPIP2 bind to RLK7. **(B)** Atom and residue parameters of AtPIP1 and AtPIP2 with RLK7 obtained from molecular docking. **(C)** Dissolution parameters of AtPIP1 and AtPIP2 with the RLK7 obtained from molecular docking.
