# Supplementary figures and images for: Structure and functional divergence of PIP peptide family revealed by functional studies on PIP1 and PIP2 in Arabidopsis thaliana
Source: Front Plant Sci. 2023 Nov 24;14:1208549. doi: 10.3389/fpls.2023.1208549 (PMC10704477; doi:10.3389/fpls.2023.1208549)

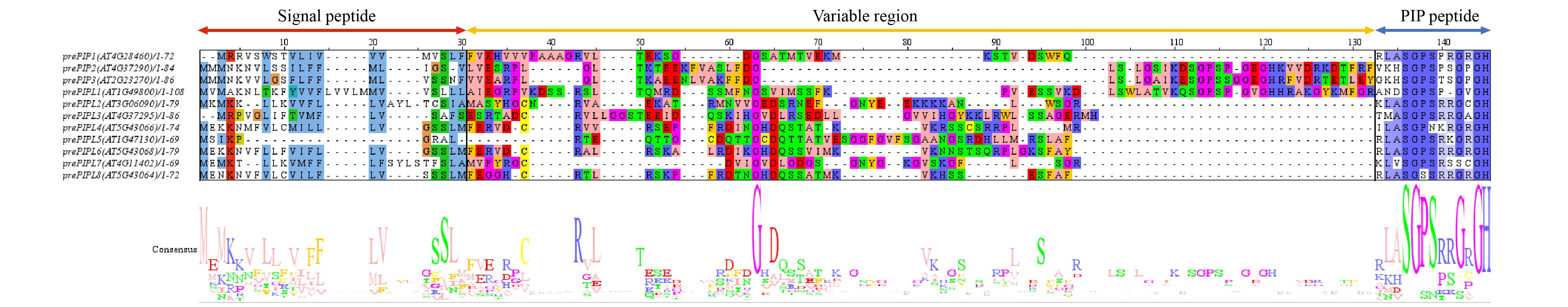

Supplement: Supplementary Figure 1 — Gene structure of 11 PIP family members in Arabidopsis. [file Image_1.jpeg]

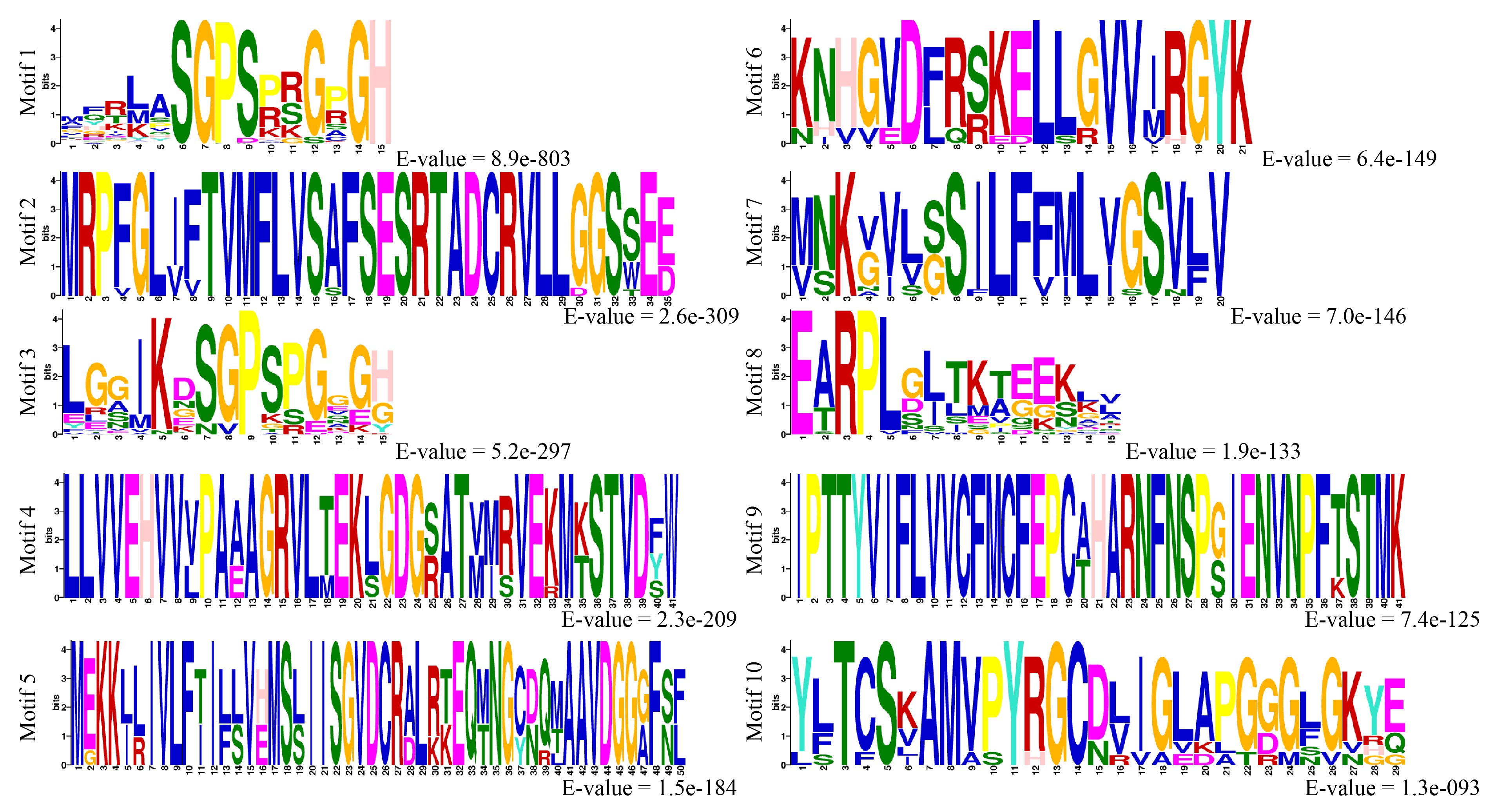

Supplement: Supplementary Figure 2 — The MEME suite was used to identify conserved motifs of 128 PIP family members. [file Image_2.jpeg]

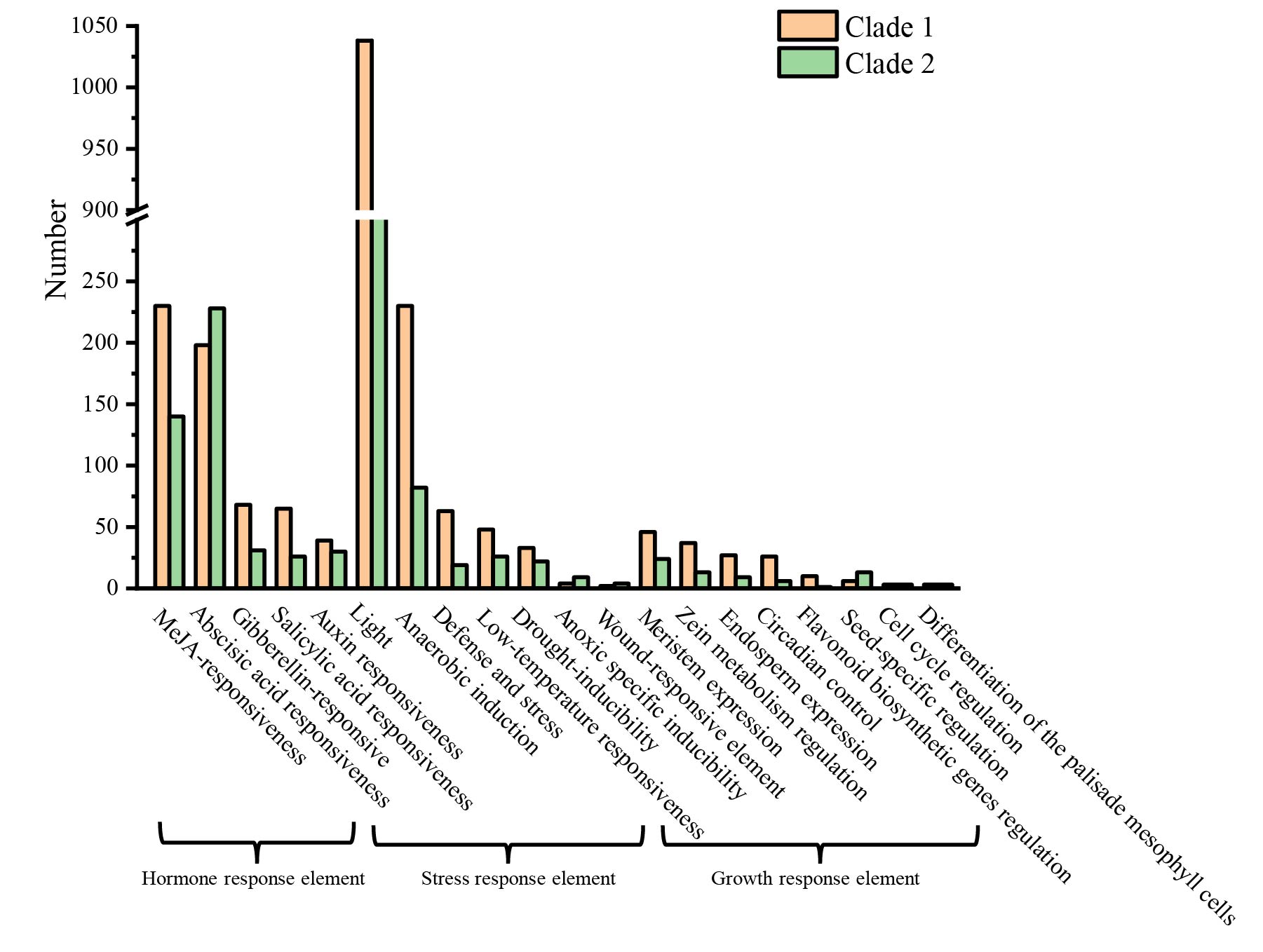

Supplement: Supplementary Figure 3 — Distribution of cis-acting elements with related functions in Clade I and Clade II. [file Image_3.jpeg]

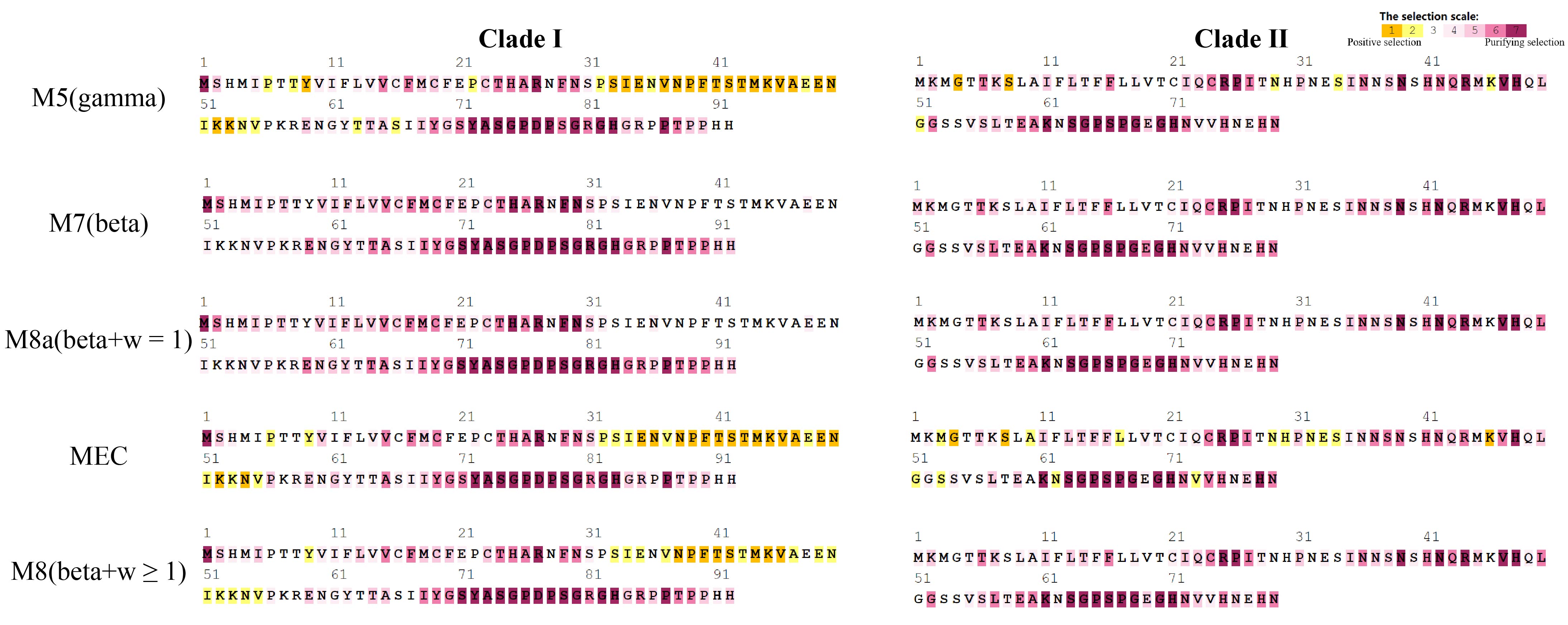

Supplement: Supplementary Figure 4 — Mutation of amino acid sites in five models were analyzed by SELECTION. [file Image_4.jpeg]

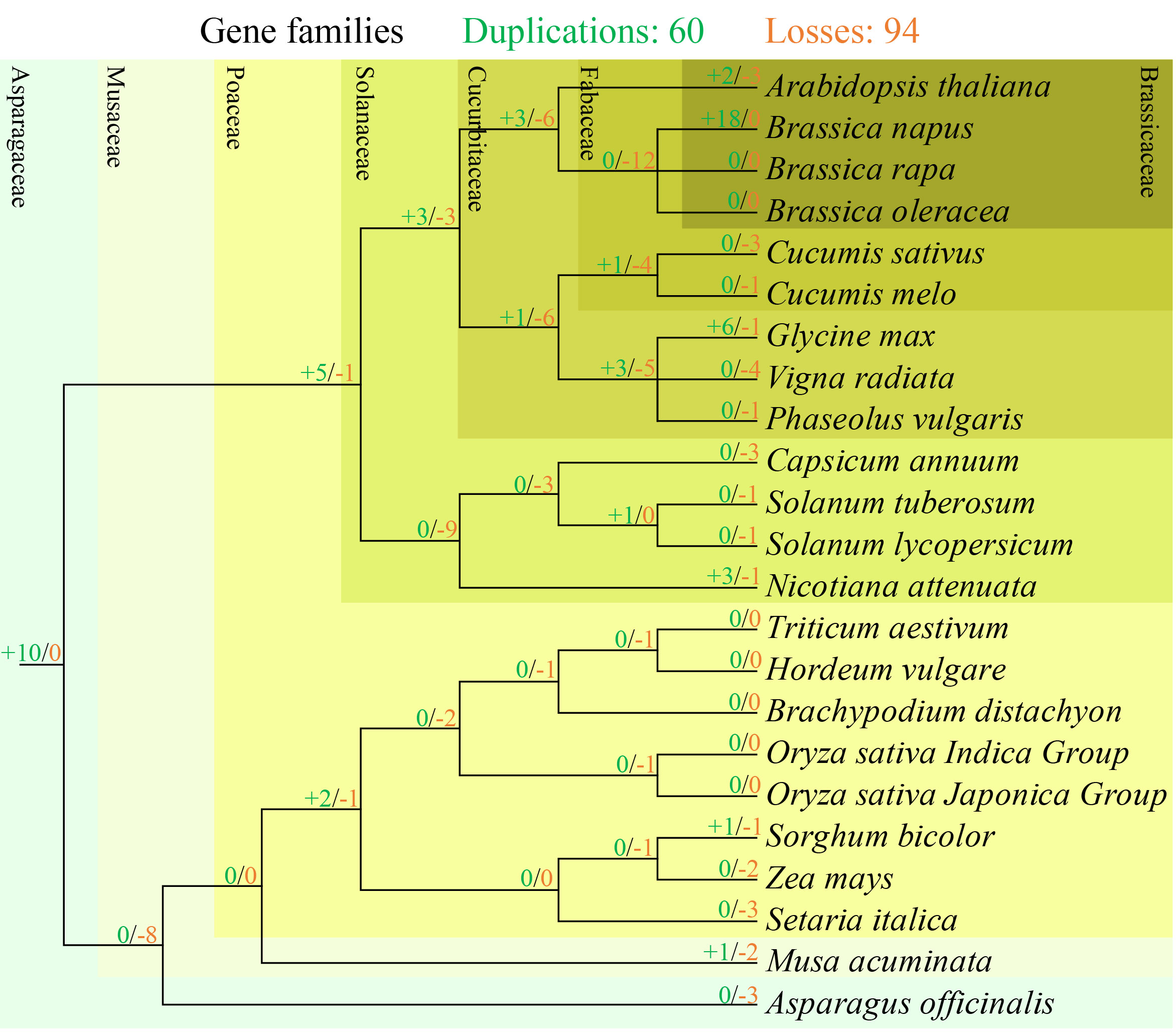

Supplement: Supplementary Figure 5 — Duplications and losses of gene families in different species. [file Image_5.jpeg]

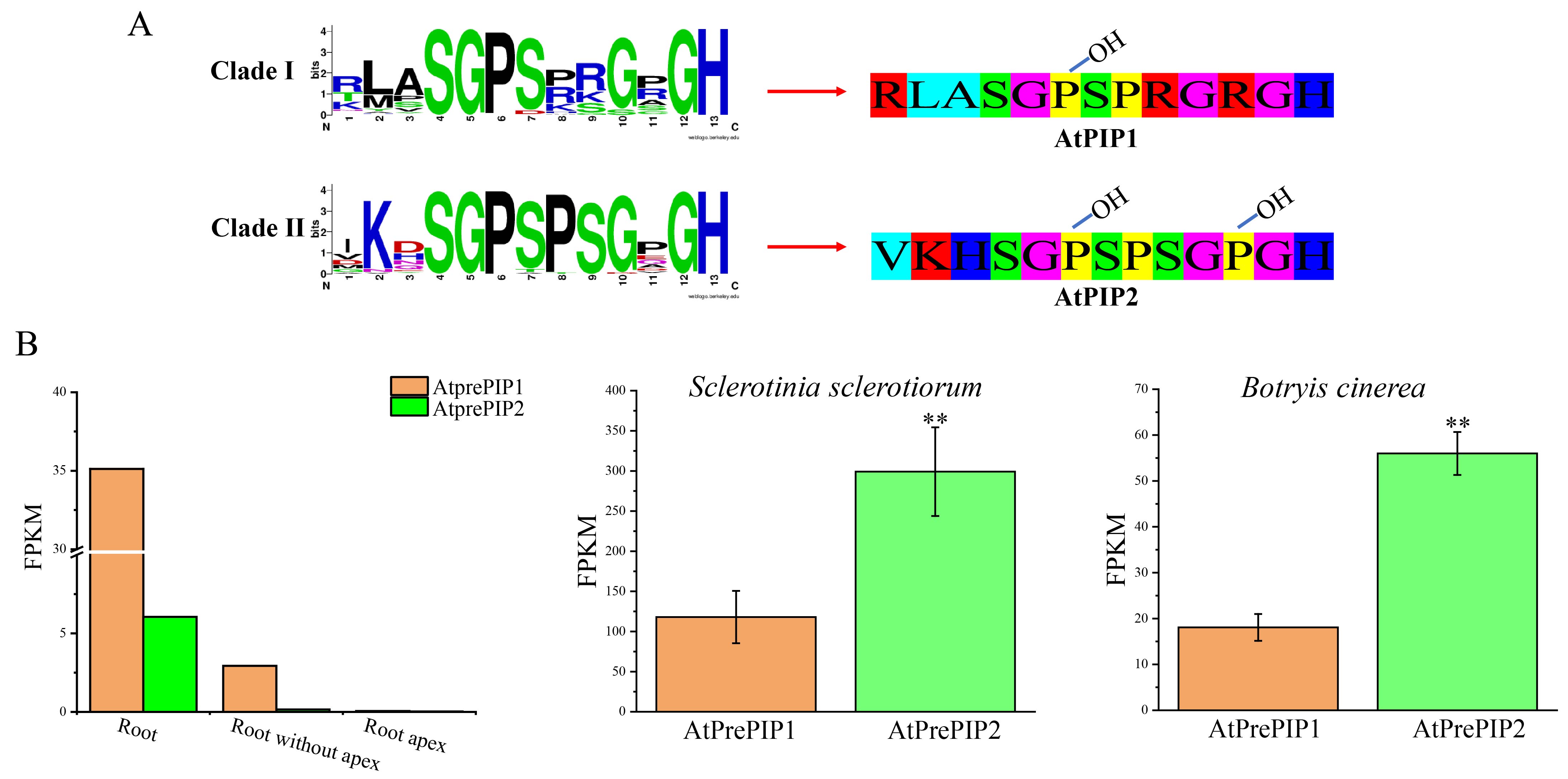

Supplement: Supplementary Figure 6 — Characteristics and precursor gene expression of AtPIP1 and AtPIP2. (A) Proportion and dominant PIPs of Clade I and Clade II. (B) Expression of AtprePIP1 and AtprePIP2 in Arabidopsis root from BAR database and after infection by Sclerotinia sclerotiorum and Botryis cinerea from GEO database. [file Image_6.jpeg]

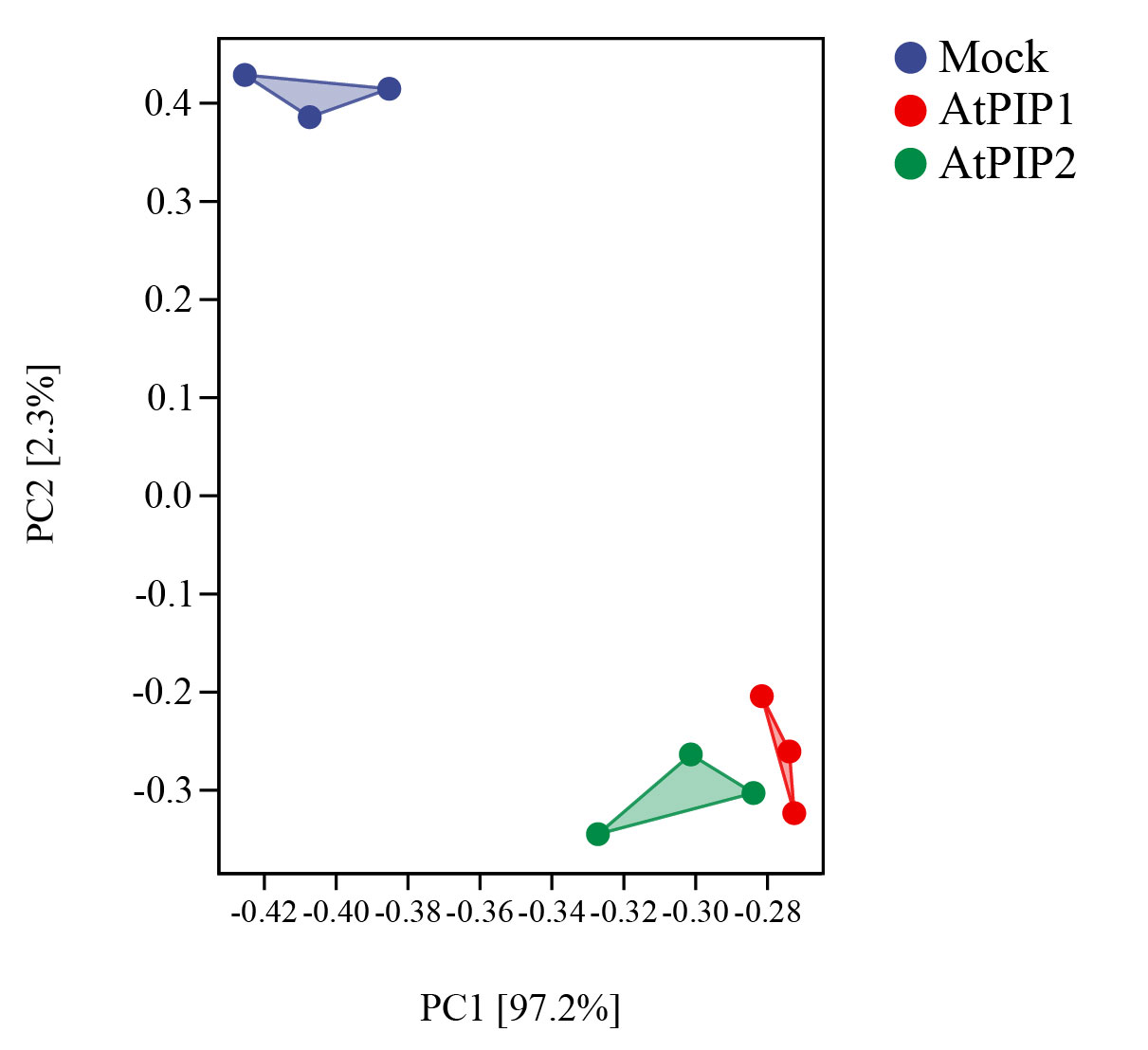

Supplement: Supplementary Figure 7 — Transcriptome analysis after 1 h of AtPIP1 and AtPIP2 treatment. (A) Principal Component Analysis (PCA) of Arabidopsis seedlings treated with AtPIP1 and AtPIP2. (B) Differences in highly expressed genes after 1 h of AtPIP1 and AtPIP2 treatment. [file Image_7.jpeg]

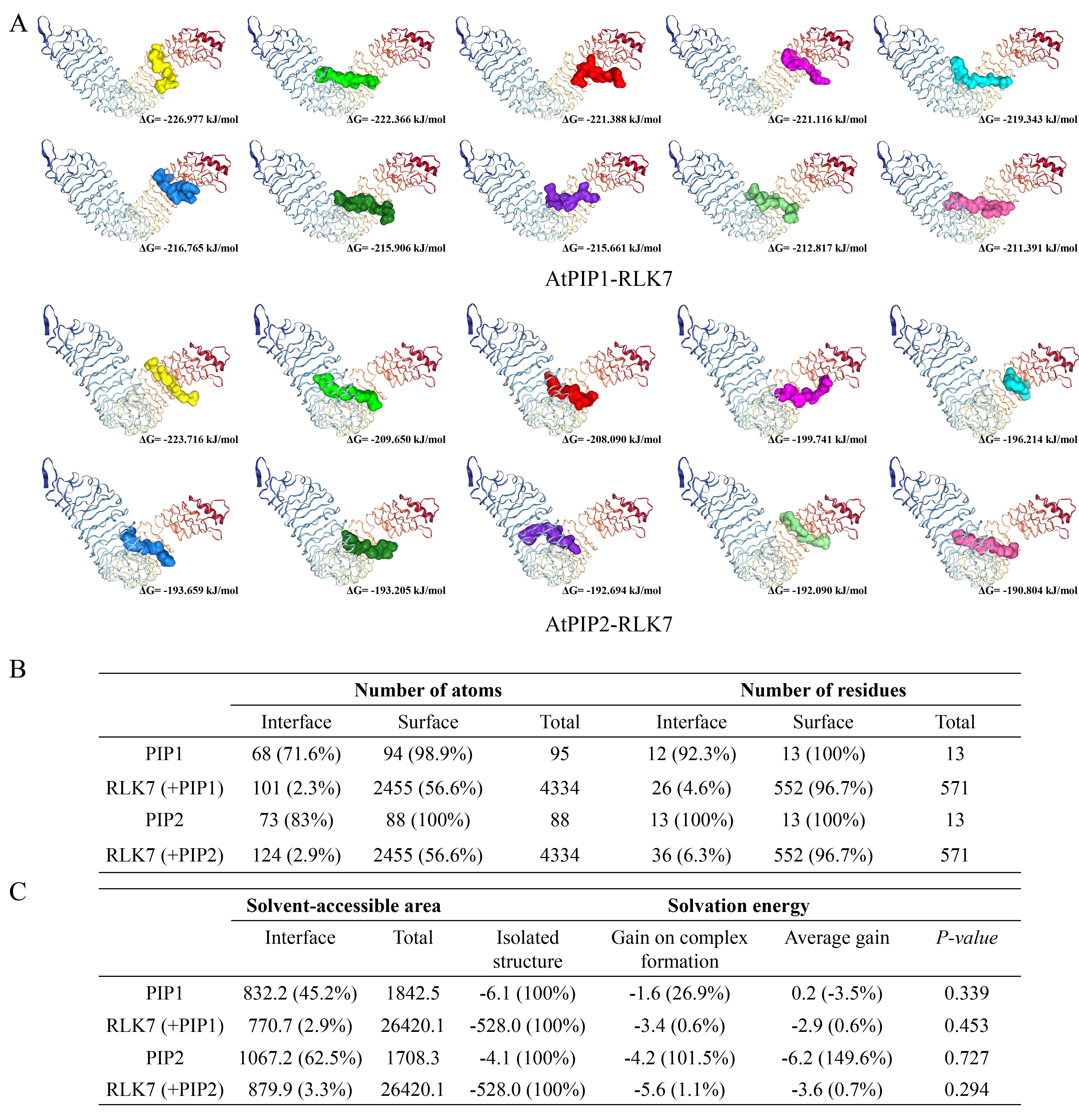

Supplement: Supplementary Figure 8 — Binding parameters of AtPIP1 and AtPIP2 with the RLK7 obtained from molecular docking. (A) The 10 most stable structures when AtPIP1 and AtPIP2 bind to RLK7. (B) Atom and residue parameters of AtPIP1 and AtPIP2 with RLK7 obtained from molecular docking. (C) Dissolution parameters of AtPIP1 and AtPIP2 with the RLK7 obtained from molecular docking. [file Image_8.jpeg]
